# Supplementary figures and images for: Critical Appraisal of Leibovich 2018 and GRANT Models for Prediction of Cancer-Specific Survival in Non-Metastatic Chromophobe Renal Cell Carcinoma
Source: Cancers (Basel). 2023 Apr 5;15(7):2155. doi: 10.3390/cancers15072155 (PMC10093654; doi:10.3390/cancers15072155)

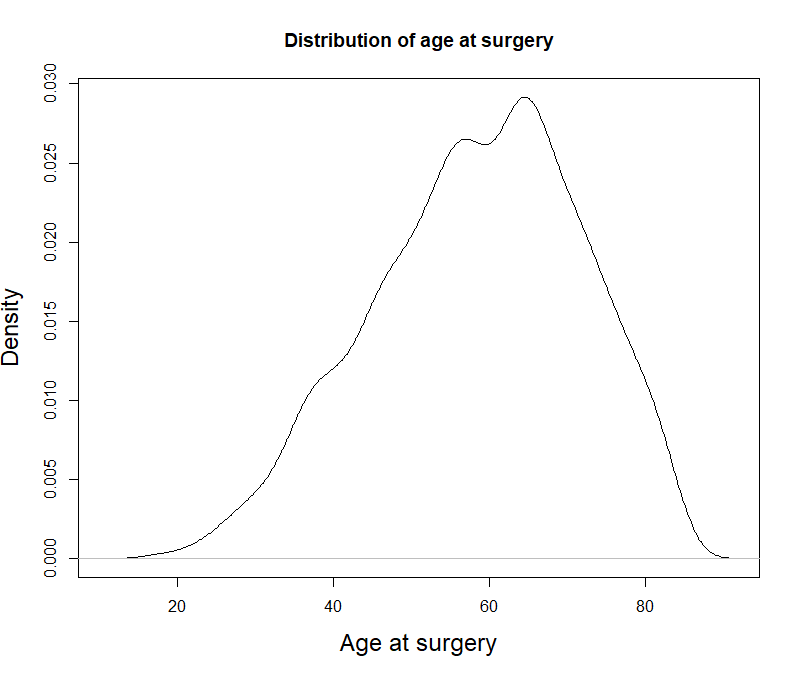

Supplement: Supplementary file 1 [file cancers-15-02155-s001.zip › Supplementary Figure S1.tiff]

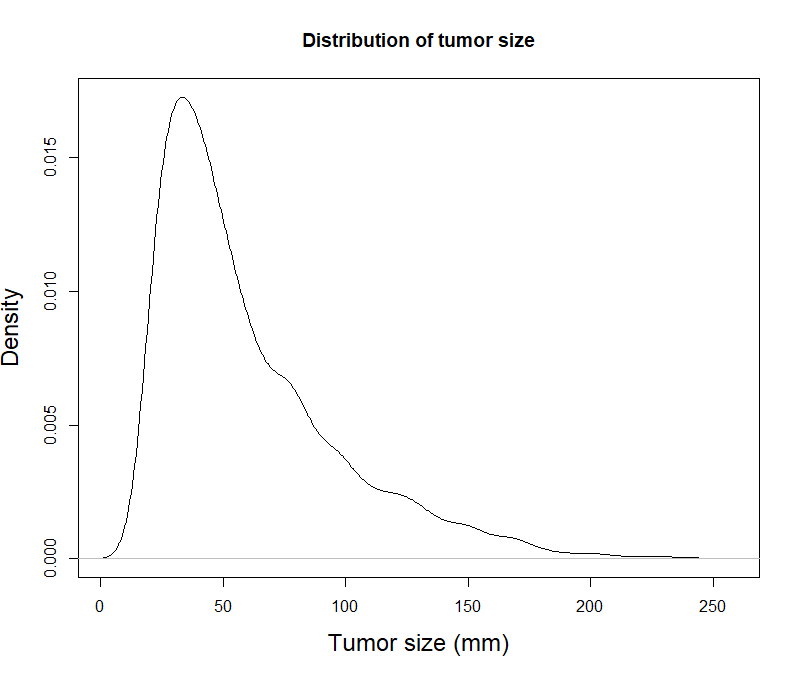

Supplement: Supplementary file 1 [file cancers-15-02155-s001.zip › Supplementary Figure S2.tiff]
